# Wissen und Haltungen zur Suizidassistenz: Befragung unter Mitgliedern der Deutschen Gesellschaft für Anästhesiologie und Intensivmedizin e. V. (DGAI)

**DOI:** 10.1007/s00101-025-01547-0

**Published:** 2025-05-16

**Authors:** Yann-Nicolas Batzler, Manuela Schallenburger, Jacqueline Schwartz, Till Brune, Susanne Feit, Theresa Tenge, Remo Küppers, Stefan Meier, Martin Neukirchen

**Affiliations:** 1 Zentrum für altersübergreifende Palliativmedizin und Kinderschmerztherapie, Universitätsklinikum des Saarlandes, Homburg/Saar, Deutschland; 2 Interdisziplinäres Zentrum für Palliativmedizin, Medizinische Fakultät und Universitätsklinikum Düsseldorf, Heinrich-Heine-Universität Düsseldorf, Moorenstr. 5, 40225 Düsseldorf, Deutschland; 3 Centrum für Integrierte Onkologie Aachen Bonn Cologne Düsseldorf (CIO ABCD), Düsseldorf, Deutschland; 4 Klinik für Anästhesiologie, Medizinische Fakultät und Universitätsklinikum Düsseldorf, Heinrich-Heine-Universität Düsseldorf, Düsseldorf, Deutschland

**Keywords:** Assistierter Suizid, Sterbehilfe, Gesetzgebung, Suizidalternativen, Palliativmedizin, Suicide assistance, Suicide alternatives, Legislature, Euthanasia, Palliative care

## Abstract

**Hintergrund:**

In Deutschland wurde 2020 das Verbot der geschäftsmäßigen Förderung der Selbsttötung durch das Bundesverfassungsgericht aufgehoben, sodass diesbezüglich in Deutschland eine der liberalsten Situationen weltweit herrscht. Eine gesetzliche Regelung ist ungewiss – zuletzt scheiterten zwei Gesetzesvorschläge im Deutschen Bundestag.

**Ziel:**

Wissen zur rechtlichen Lage in Deutschland sowie Haltungen zur Suizidassistenz unter Mitgliedern der Deutschen Gesellschaft für Anästhesiologie und Intensivmedizin (DGAI) sollen abgebildet werden.

**Material und Methoden:**

Von der Studiengruppe wurde ein Online-Fragebogen entwickelt. Dieser enthielt Fragen zu Demografie, zur (standes-)rechtlichen Situation der Suizidassistenz in Deutschland sowie zur persönlichen Haltung diesbezüglich. Er wurde via Mitglieder-Newsletter verteilt.

**Ergebnisse:**

Von 25.573 via E‑Mail erreichbaren DGAI-Mitgliedern nahmen 686 bis zum Ende teil. Davon waren 99 % (*n* = 676/686) Ärzt:innen. Es nahmen 86 % (*n* = 589/686) fälschlicherweise an, Suizidassistenz dürfe in Deutschland nicht geschäftsmäßig angeboten werden; 77 % (*n* = 527/686) wünschten sich eine gesetzliche Regelung. Von den Befragten waren 55 % (*n* = 374/686) der Ansicht, dass Gesundheitsmitarbeitende die richtigen Ansprechpartner sind, um über die Zulässigkeit eines assistierten Suizids zu entscheiden; 23 % (*n* = 159/686) sprachen Patient:innen noch nie auf Todeswünsche an. Eine Mitwirkung beim assistierten Suizid lehnten 71 % (*n* = 486/686) bei Patient:innen unabhängig des Gesundheitszustands ab, 65 % (*n* = 443/686) konnten sich diese nur im palliativen Setting vorstellen.

**Diskussion:**

Zahlreiche Teilnehmer:innen wiesen Wissenslücken zur gültigen Rechtslage bezüglich der Suizidassistenz auf. Eine gesetzliche Regulierung soll Rechtssicherheit schaffen. Die Ergebnisse unterstreichen die Komplexität des Themas um den assistierten Suizid.

**Graphic abstract:**

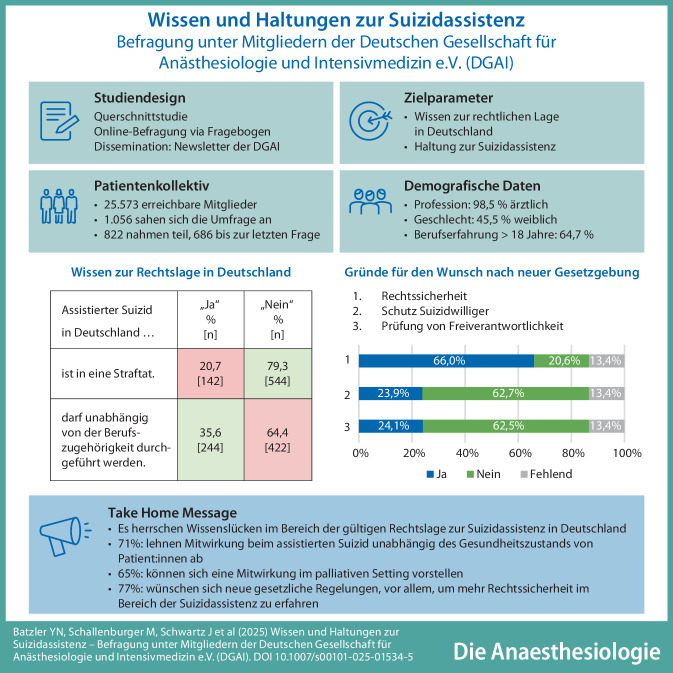

## Abstract (en)

**Background:**

The ban on assisted suicide “intended as a recurring pursuit” was lifted by the German Federal Constitutional Court in 2020. Consequently, the German Medical Council repealed § 16 sentence 3 of the (model) professional code (“They [doctors] may not provide assistance for suicide”). Two legislative proposals to regulate assisted suicide failed in the German Bundestag in 2023. To date, medical staff lack a basis for action when dealing with patients who wish to die. The opinions, attitudes and perspectives of professional groups are important with regard to assisted suicide in order to be able to draw up practical regulations that can be implemented in everyday medical practice.

**Objectives:**

The aim of this survey is to assess the knowledge about the legal situation in Germany as well as perceptions and attitudes towards assisted suicide among members of the German Society of Anaesthesiology and Intensive Care Medicine (DGAI).

**Materials and methods:**

The study group developed a questionnaire in repeated interdisciplinary and multiprofessional discussions based on a literature review and a previous survey among younger physicians. The questionnaire consisted of questions on the legal and statutory situation of assisted suicide in Germany, personal attitudes and demographic data. It was distributed via the DGAI member newsletter using an online link.

**Results:**

From 08/2023–01/2024, out of 25,573 reachable members, 1056 viewed the survey (view rate: 4%), 822 participated, of which 686 completed the questionnaire (completion rate: 83%). Of all participants, 99% (*n* = 676/686) were physicians; 56% (*n* = 385/686) were older than 50 years. 45% (*n* = 310/686) of participants stated that they were aware of the judgment on the invalidity of section 217 of the German criminal code. 86% (*n* = 589/686) of respondents incorrectly believed that it is prohibited in Germany to offer assisted suicide intended as a recurring pursuit and 21% (*n* = 142/686) of respondents falsely believed that assisted suicide is a criminal offense in Germany. 77% (*n* = 527/686) would prefer a new legal regulation on assisted suicide in order to achieve greater legal certainty. 55% (*n* = 374/686) stated that healthcare workers are the appropriate point of contact to decide on the permissibility of assisted suicide. Of all respondants, 45% (*n* = 305/686) have asked patients about the existence of death wishes in their professional career; 23% (*n* = 159/686) of respondents have never done so. 71% (*n* = 486/686) declined to participate in assisted suicide for patients regardless of the health condition; 65% (*n* = 443/686) indicated they could only envision participation in a palliative care setting.

**Conclusions:**

Participating members of the DGAI show a lack of knowledge regarding the current legal framework surrounding assisted suicide in Germany. They express the hope that future legal regulations will provide legal certainty in this sensitive area. The findings of this study highlight the complexity inherent in the issue of assisted suicide. Subsequent research and policy development should focus on addressing the needs and challenges faced by clinicians and patients, while fostering the establishment of clear guidelines and regulatory frameworks to ensure the provision of safe and ethically responsible care.

## Hinführung zum Thema

In Deutschland wurde 2020 das Verbot der geschäftsmäßigen Förderung der Selbsttötung durch das Bundesverfassungsgericht aufgehoben. Seitdem herrscht in Deutschland gesetzlich eine der liberalsten Situationen weltweit. Eine Regulierung wurde dem Gesetzgeber überlassen. Bisher scheiterten zwei Gesetzesentwürfe im Deutschen Bundestag. Die Haltung von medizinischem Personal sollte im Rahmen der Diskussion Beachtung finden. Um einen Beitrag für eine mögliche Jurisdiktion zu leisten, wurden unter Mitgliedern der Deutschen Gesellschaft für Anästhesiologie und Intensivmedizin e. V. (DGAI) das Wissen zur (standes-)rechtlichen Situation sowie Haltungen zur Suizidassistenz untersucht.

## Hintergrund und Fragestellung

Medizinisches Personal wird unabhängig von Fachbereich oder Profession häufig mit Todeswünschen und gelegentlich mit dem Wunsch nach assistiertem Suizid konfrontiert [1–4]. Eine Auseinandersetzung mit der rechtlichen Situation bezüglich der Suizidassistenz in Deutschland sowie die Ausbildung einer eigenen Haltung zum Thema sind daher unerlässlich.

Im Februar 2020 erklärte das Bundesverfassungsgericht § 217 des Strafgesetzbuches (StGB), welcher die geschäftsmäßige Förderung der Selbsttötung strafrechtlich belangte, für „nichtig“, da „Staat und Gesellschaft“ das Recht auf Suizid, auch unter Mithilfe Dritter, als „Akt autonomer Selbstbestimmung zu respektieren“ haben [5]. Nach diesem Urteil hob der Deutsche Ärztetag § 16 Satz 3 der (Muster‑)Berufsordnung („Sie [Ärztinnen und Ärzte] dürfen keine Hilfe zur Selbsttötung leisten.“) auf [6]. 2023 scheiterten zwei Gesetzesentwürfe zur Regelung der Suizidassistenz im Deutschen Bundestag; ein Entwurf zur Stärkung suizidpräventiver Maßnahmen wurde angenommen [7].

Todeswünsche sind im Rahmen von lebenslimitierenden Erkrankungen häufig Teil natürlicher Ambivalenz [4], ohne dass ein Handlungsdruck entsteht. Da v. a. medizinisches Personal eine entscheidende Rolle spielt, Patient:innen mit Todeswünschen zu beraten und zu betreuen, sollte ein fundiertes Wissen bestehen. Zur möglichen Regulierung der Suizidassistenz in Deutschland sind Meinungen und Perspektiven von medizinischem Personal relevant, um praxisnahe und im medizinischen Alltag umsetzbare Reglementierungen zu verfassen. Befragungen hierzu erfolgten bereits unter Mitgliedern der Deutschen Gesellschaft für Psychiatrie und Psychotherapie, Psychosomatik und Nervenheilkunde e. V. (DGPPN), unter jungen Ärzt:innen sowie unter Mitgliedern der Deutschen Gesellschaft für Hämatologie und Medizinische Onkologie e. V. (DGHO) [3, 8, 9].

Die vorliegende Befragung schließt sich an die bereits erfolgten an. Sie hat das Ziel, Wissen zur rechtlichen Lage in Deutschland sowie Haltungen zur Suizidassistenz unter Mitgliedern der Deutschen Gesellschaft für Anästhesiologie und Intensivmedizin e. V. (DGAI) abzubilden.

## Studiendesign und Untersuchungsmethoden

Die Studiengruppe entwickelte anhand von Literatur einen Fragebogen, welcher in einer Expertengruppe erweitert und editiert wurde. Der Fragebogen wurde erstmalig in einer Befragung zur Suizidassistenz unter jungen Mediziner:innen verwendet [8]. Er fand zudem Verwendung im Rahmen einer Befragung unter Mitgliedern der Deutschen Gesellschaft für Palliativmedizin (DGP) [9]. Auf Grundlage dieser Erhebungen wurden Fragen für die Zielgruppe der DGAI im Rahmen eines wiederholten Austausches in einer interdisziplinären und multiprofessionellen Expertengruppe, bestehend aus Mediziner:innen und Pflegenden aus dem Bereich der Anästhesie, Intensivmedizin und Palliativmedizin, aktualisiert: Unklar formulierte Fragen, v. a. zur Einschätzung professionsbezogener Verantwortlichkeit, wurden angepasst und zur Überprüfung des Wissens nach diesbezüglicher Selbsteinschätzung Fragen zur Rechtslage eingefügt.

Der Fragebogen bestand aus Fragen zur Demografie, zur (standes-)rechtlichen Situation der Suizidassistenz in Deutschland und persönlichen Einstellungen. Demografische Daten wurden anhand von Fragen mit Mehrfachantworten in Kategorien erfasst. Zur Bestimmung von Häufigkeiten wurden Abstufungen verwendet: „noch nie“ (*n* = 0), „vereinzelt“ (*n* ≤ 2), „gelegentlich“ (*n* = 3–10), „häufig“ (*n* = 11–50) und „sehr häufig“ (*n* > 50). Daten zu Zustimmung bzw. Ablehnung wurden anhand einer Fünf-Punkt-Likert-Skala erhoben, wobei „trifft voll zu“ und „trifft eher zu“ als Zustimmung gewertet wurden. Es war teilweise möglich, Fragen mit „keine Angabe“ zu beantworten und Fragen zu überspringen. Der Fragebogen wurde auf der Plattform Unipark (https://www.unipark.com) der Tivian XI GmbH (Köln, Deutschland) eingestellt. Die Darstellung der Ergebnisse folgt der CHERRIES-Checkliste [10]. Es erfolgte eine deskriptive Auswertung, Interferenzanalysen wurden mittels Cramers V auf Basis von χ^2^-Tests sowie Spearman- und Pearson-Korrelationen durchgeführt. Nur die Fragebogen, die bis zur letzten Frage ausgefüllt wurden, wurden ausgewertet. Fehlende Daten wurden in die Analyse mitaufgenommen. Die Datenauswertung erfolgte mit Microsoft Excel 365 (Version 16.78, 10/2023, Microsoft Inc., Redmond, WA, USA) und JASP (Version 0.18.3, 01/2024). Die Studie wurde durch die Ethikkommission der Heinrich-Heine-Universität Düsseldorf positiv votiert (Studien-Nr.: 2022-2193_3 vom 07.08.2023).

## Ergebnisse

Der Umfrage-Link wurde im Newsletter der DGAI im August 2023 an 25.573 Mitglieder (inkl. erreichbare Mitglieder des Berufsverbands Deutscher Anästhesisten e. V., BDA) via E‑Mail verschickt. Eine Erinnerung an die Umfrage wurde im Oktober 2023 auf gleichem Wege verschickt. Von August 2023 bis Januar 2024 sahen sich 1056 Mitglieder die Umfrage an („view rate“: 4 %). 822 davon nahmen teil („participation rate“: 78 %), 686 füllten den Fragebogen bis zum Ende aus („completion rate“: 83 %).

### Demografie

98,5 % (*n* = 676) der Teilnehmer:innen waren Ärzt:innen, 0,3 % Pflegende (fehlend: *n* = 8). Dem weiblichen Geschlecht fühlten sich 45,5 % (*n* = 312) zugehörig (Tab. 1). 64,7 % (*n* = 444) hatten mehr als 18 Jahre Berufserfahrung, 2,3 % (*n* = 16) maximal 4 Jahre.

**Tab. 1:** Demografische Daten der Teilnehmer:innen (*n* = 686); verfügbare Daten der Mitgliederstruktur der Deutschen Gesellschaft für Anästhesiologie und Intensivmedizin (DGAI) und des Berufsverbands Deutscher Anästhesistinnen und Anästhesisten (BDA); Geschlechterverteilung in der Bundesrepublik Deutschland (BRD) 2022

	Teilnehmer:innen (DGAI) *n* [%]	DGAI und BDA [%]	Bevölkerung BRD [%]
*Geschlecht*			
Weiblich	312 [45,5]	[ca. 34]	[50,7]
Männlich	353 [51,5]	n. b.	[49,2]
Divers	1 [0,1]	n. b.	[< 0,1]
Keine Angabe	13 [1,9]	n. b.	n. b.
Fehlende Daten	7 [1,0]	n. b.	n. b.
*Alter (Jahre)*			
< 30	10 [1,5]	n. b.	n. b.
31–40	111 [16,1]	n. b.	n. b.
41–50	168 [24,5]	n. b.	n. b.
51–60	192 [28,0]	n. b.	n. b.
> 60	193 [28,2]	n. b.	n. b.
Keine Angabe	5 [0,7]	n. b.	n. b.
Fehlend	7 [1,0]	n. b.	n. b.
*Facharztstatus*			
Ja	613 [89,4]	[ca. 75,0]	n. b.
Nein	63 [9,2]	[ca. 25,0]	n. b.
Fehlend	10 [1,5]	n. b.	n. b.

*n.b.* nicht bekannt

### Wissen zur (standes-)rechtlichen Lage in Deutschland

In der Selbsteinschätzung gaben 89,2 % (*n* = 612/686) der Befragten an, die Unterschiede zwischen den Begrifflichkeiten „freiwilliger Verzicht auf Essen und Trinken (FVET)“, „assistierter Suizid“ und „Tötung auf Verlangen“ (in Deutschland strafbar) zu kennen.

Jeweils 45,2 % (*n* = 310/686) der Teilnehmer:innen gaben an, das Urteil zur Nichtigkeit von § 217 sowie die im Sommer 2023 abgelehnten Gesetzesentwürfe zu kennen. In etwa genauso viele gaben an, diese nicht zu kennen (45 %, *n* = 309/686 bzw. 44,8 %, *n* = 307/686). Es fand sich ein schwacher Zusammenhang mit dem Facharztstatus (Cramers V = 0,18, *p* < 0,001). 85,9 % (*n* = 589/686) der Befragten waren fälschlicherweise der Annahme, der assistierte Suizid dürfe in Deutschland nicht geschäftsmäßig angeboten werden. Unter den Befragten waren 20,7 % (*n* = 142/686) fälschlicherweise der Annahme, der assistierte Suizid sei in Deutschland eine Straftat. Weitere Ergebnisse sind in Tab. 2 zusammengefasst.

**Tab. 2:** Angaben zum Wissen zur aktuell gültigen (standes-)rechtlichen Lage in Deutschland (*n* = 686)

Assistierter Suizid …	Ja *n* [%]	Nein *n* [%]
– ist in Deutschland eine Straftat (Richtig: Nein)	142 [20,7]	544 [79,3]
– darf in Deutschland geschäftsmäßig angeboten werden (Richtig: Ja)	97 [14,1]	589 [85,9]
– ist in Deutschland den Ärzt:innen standesrechtlich verboten (Richtig: Nein)	257 [37,5]	429 [62,5]
– darf in Deutschland unabhängig von der Berufszugehörigkeit durchgeführt werden (Richtig: Ja)	244 [35,6]	442 [64,4]

### Regulierung der Suizidassistenz

**Abb. 1:**
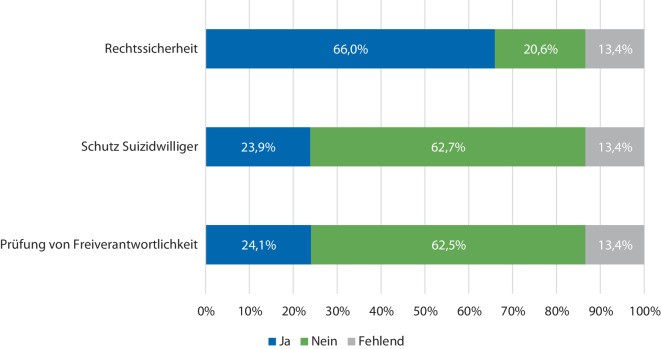
Gründe für den Wunsch nach neuer Gesetzgebung zum assistierten Suizid

Unter der Teilnehmer:innen wünschten sich 76,8 % (*n* = 527/686) eine neue gesetzliche Regelung zur Suizidassistenz, v. a., um hierdurch mehr Rechtssicherheit zu erfahren (66 %, *n* = 453/686, fehlend: *n* = 92). (Abb. 1).

### Erfahrungen mit dem Wunsch nach Suizidassistenz

39,1 % (*n* = 268/686) der Teilnehmer:innen hatten in ihrer medizinischen Laufbahn bereits mehr als 10 Patient:innen, die einen Todeswunsch äußerten, betreut. 44,5 % (*n* = 305/686) der Befragten hatten in ihrer beruflichen Laufbahn vereinzelt bis gelegentlich Patient:innen auf das Vorhandensein von Todeswünschen angesprochen, 23,2 % (159/686) der Befragten noch nie. Es bestand ein schwach positiver Zusammenhang zwischen Berufserfahrung und der Frequenz des Ansprechens auf Todeswünsche (Cramers V = 0,11, *p* = 0,006). Die wenigsten der Teilnehmer:innen wurden bereits häufig bis sehr häufig um eine Mithilfe beim FVET und assistierten Suizid gebeten (Tab. 3). 94,3 % (*n* = 647/686) der Befragten wirkten bis dato noch nie bei einem assistierten Suizid mit, 5,7 % (*n* = 39/686) taten dies bereits.

**Tab. 3:** Ansprechen von Todeswünschen und Bitte um Mithilfe beim freiwilligen Verzicht auf Essen und Trinken (FVET) und assistiertem Suizid (*n* = 686)

	Noch nie *n* [%]	Vereinzelt (≤ 2) *n* [%]	Gelegentlich (3–10) *n* [%]	Häufig (11–50) *n* [%]	Sehr häufig (> 50) *n* [%]	Fehlend *n* [%]
Ich habe Patient:innen aktiv auf das Vorhandensein von Todeswünschen angesprochen	159 [23,2]	140 [20,4]	165 [24,1]	131 [19,1]	82 [12,0]	9 [1,3]
Ich bin um Mithilfe beim FVET gebeten worden	387 [56,4]	121 [17,6]	88 [12,8]	58 [8,5]	22 [3,2]	10 [1,5]
Ich bin um Mithilfe beim assistierten Suizid gebeten worden	336 [49,0]	200 [29,2]	92 [13,4]	32 [4,7]	15 [2,2]	11 [1,6]

### Professionsbezogene Zuständigkeiten

Es waren 54,5 % (*n* = 374/686) der Teilnehmer:innen der Ansicht, Mitarbeitende im Gesundheitswesen seien die richtigen Ansprechpartner:innen, um im Einzelfall über die Zulässigkeit eines assistierten Suizids zu entscheiden, 28,1 % (*n* = 193/686) waren dahingehend unentschlossen („weder noch“). Bezüglich der unmittelbaren Mitwirkung wurden mögliche Zuständigkeiten professionsbezogen auf den Berufsstand der Pflegenden und Ärzt:innen erfragt (Tab. 4). Hierbei herrschte v. a. dahingehend Zustimmung, dass eine Mitwirkung eine ärztliche Aufgabe sein kann (69,4 %; *n* = 476/686). Eine definitive Zuweisung zur ärztlichen Profession war für 43 % immanent. Die Einschätzung, dass die Mitwirkung bei einem assistierten Suizid ärztliche Aufgabe ist, korrelierte schwach positiv mit Berufserfahrung (r = 0,13, *p* < 0,001) und Alter (r = 0,14, *p* < 0,001) der Teilnehmer:innen.

**Tab. 4:** Professionsbezogene Mitwirkung beim assistierten Suizid (*n* = 686)

Die unmittelbare Mitwirkung beim assistierten Suizid …	Trifft voll zu *n* [%]	Trifft eher zu *n* [%]	Weder noch *n* [%]	Trifft eher nicht zu *n* [%]	Trifft gar nicht zu *n* [%]	Fehlend *n* [%]
– *kann* eine ärztliche Aufgabe sein	168 [24,5]	308 [44,9]	79 [11,5]	70 [10,2]	55 [8,0]	6 [0,9]
– *ist* eine ärztliche Aufgabe	97 [14,1]	198 [28,9]	142 [20,7]	119 [17,3]	123 [17,9]	7 [1,0]
– *kann* eine pflegerische Aufgabe sein	53 [7,7]	221 [32,2]	102 [14,9]	170 [24,8]	128 [18,7]	12 [1,7]
– *ist* eine pflegerische Aufgabe	16 [2,3]	85 [12,4]	148 [21,6]	195 [28,4]	233 [34,0]	9 [1,3]

### Assistierter Suizid und Gesundheitszustand von Patient:innen

**Abb. 2:**
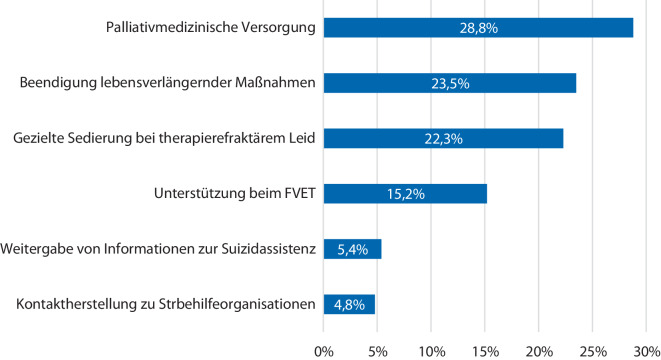
Mögliche suizidpräventive Maßnahmen aus Sicht der Teilnehmer:innen. (Mehrfachantworten möglich, *n* = 855 abgegebene Antworten). *FVET* freiwilliger Verzicht auf Essen und Trinken

Unter den Teilnehmer:innen lehnten 70,9 % (*n* = 486/686, fehlend: *n* = 7) eine Mitwirkung bei einem assistierten Suizid bei Patient:innen unabhängig des Gesundheitszustands ab. Für diese Einschätzung ergab sich ein signifikanter Zusammenhang mit dem Facharztstatus (χ^2^ = 20,18, *p* < 0,001). Für Patient:innen, die sich in einer palliativen Behandlungssituation befinden, lag die Ablehnung einer Mitwirkung bei 26,3 % (*n* = 180/686, fehlend: *n* = 9) und 64,6 % (*n* = 443/686) der Teilnehmer:innen gaben an, sich eine Mitwirkung nur im palliativen Setting vorstellen zu können. Beim Wunsch nach assistiertem Suizid lehnten 61,5 % (*n* = 422/686) der Befragten eine Beschränkung auf suizidpräventive Maßnahmen ab. Bezüglich der Maßnahmen der Suizidprävention waren Mehrfachantworten möglich. Es wurden insgesamt 855 Antworten abgegeben. Allen voran wurden eine palliativmedizinische Versorgung als wichtige suizidpräventive Maßnahme angesehen (Abb. 2).

## Diskussion

Die vorliegende Studie untersuchte das Wissen und die Haltung von Mitgliedern der DGAI bezüglich des assistierten Suizids. Weiterhin wurden ethische und rechtliche Aspekte dieses sensiblen Themas tiefergehend exploriert. Diese Studie erweitert das Meinungsbild zum assistierten Suizid, das bereits durch andere Umfragen zu dieser Thematik aufgezeichnet werden konnte.

### Wissen und Jurisdiktion

Trotz einer hohen Selbsteinschätzung des Wissens über verschiedene Formen des assistierten Suizids bestehen unter den Teilnehmer:innen noch immer Fehlinformationen, insbesondere bezüglich aktueller rechtlicher Entwicklungen. Diese Feststellung deckt sich mit Erkenntnissen einer Befragung unter jungen Mediziner:innen, bei der 71 % der Teilnehmer:innen die Inhalte des Beschlusses des Bundesverfassungsgerichts zur Nichtigkeit von § 217 und 72 % die im Sommer 2023 vorgeschlagenen Gesetzesentwürfe zur Regulierung der Suizidassistenz nicht kannten [8]. In unserer Befragung zeigte sich ein schwacher, aber signifikanter Zusammenhang mit vorliegendem Facharztstatus, wobei die Literatur dies kontrovers beleuchtet [8, 11, 12].

76 % der Teilnehmer:innen dieser Befragung sprachen sich für eine neue gesetzliche Regulierung der Suizidassistenz aus. Dies deckt sich mit den Ergebnissen einer Befragung unter Mitgliedern der DGPPN, bei der 88 % eine neue Regulierung wünschten, wenn auch die Rücklaufquote dieser Befragung deutlich über der unseren lag und eine Vergleichbarkeit so nur eingeschränkt möglich ist [8]. In der Kohorte der DGAI stand v. a. der Wunsch im Vordergrund, mehr Rechtssicherheit zu erfahren. Klar definierte gesetzliche Rahmenbedingungen könnten helfen, ethische Dilemmata zu bewältigen und eine angemessene Versorgung von Patient:innen zu gewährleisten. Die Bereitstellung klarer Richtlinien und die Schaffung eines rechtlichen Rahmens, der die Autonomie von Patient:innen respektiert, könnten dazu beitragen, die Handlungssicherheit von medizinischem Personal zu erhöhen [13, 14].

### Professionsbezogene Zuständigkeiten und Legitimation der Suizidassistenz

Die professionsbezogene Zuständigkeit beim assistierten Suizid bleibt weiterhin unklar. Da in dieser Kohorte vornehmlich Ärzt:innen teilnahmen, zeigt diese Befragung ein professionsbezogenes Meinungsbild auf. 35,2 % der Teilnehmer:innen waren der Meinung, dass eine Mitwirkung beim assistierten Suizid keine ärztliche Aufgabe ist. Unter Teilnehmer:innen der Befragung der DGPPN waren 38 % der gleichen Auffassung. Dies deckt sich mit dem Meinungsbild der Bundesärztekammer, die 2021 klar definierte, dass eine „Hilfe zur Selbsttötung“ keine ärztliche Aufgabe sei [6]. Die Option der Teilhabe wurde von knapp 70 % der Teilnehmer:innen der DGAI im Bereich der ärztlichen Zuständigkeit gesehen, von knapp 40 % im pflegerischen Zuständigkeitsbereich. Die Uneinigkeit unter den Teilnehmer:innen kann auf die Notwendigkeit klarer Richtlinien und einer kontinuierlichen interprofessionellen Kommunikation hinweisen. Die Schaffung eines kollaborativen Entscheidungsrahmens zwischen medizinischen Professionen kann dazu beitragen, Unsicherheiten zu verringern und die Versorgung von Patient:innen zu verbessern [15, 16].

Im Gegensatz dazu sind Teilnehmer:innen verschiedener medizinischer Fachgesellschaften der einheitlichen Meinung, dass die Suizidassistenz erkrankungsbezogen ihre Legitimation findet. Unter den teilnehmenden Mitgliedern der DGAI lehnten 70 % eine Mitwirkung beim assistierten Suizid unabhängig vom Gesundheitszustand ab, etwa 65 % können sich eine Mitwirkung nur im palliativen Setting vorstellen. Dies deckt sich mit Daten der Befragung unter Mitgliedern der DGPPN, unter denen v. a. „hoher Leidensdruck“ und ein „nahes Lebensende“ Voraussetzungen für eine Akzeptanz eines assistierten Suizids schafften. Die Erkrankungssituation von Patient:innen beeinflusst die Haltung zur Suizidassistenz, welche patientenzentriert definiert wird. Diese Erkenntnis sollte Grundlage möglicher Gesetze zur Suizidassistenz sein.

### Persönliche Erfahrungen und suizidpräventive Maßnahmen

Nur ca. 40 % der Teilnehmer:innen wurden in ihrer Laufbahn häufig mit Patient:innen, die einen Todeswunsch von sich aus äußerten, konfrontiert. Weiterhin wurde trotz zumeist langjähriger Berufserfahrung etwa die Hälfte der Teilnehmer:innen noch nie um Mithilfe bei einem assistierten Suizid gebeten. Aufgrund der geringen Rücklaufquote sind diese Daten allerdings nur exemplarisch zu werten. In einer Befragung unter Mitgliedern der DGHO gaben 57,1 % der Teilnehmer:innen an, bereits von Patient:innen um Informationen zum assistierten Suizid gebeten worden zu sein [3]. Gemeinsam haben beide Studien, dass im Bereich suizidpräventiver Maßnahmen v. a. eine palliativmedizinische Begleitung als Grundstein angesehen wird. Viele der Teilnehmer:innen haben noch nie bzw. nur vereinzelt Patient:innen auf das Vorhandensein von Todeswünschen angesprochen. In anderen Studien konnte gezeigt werden, dass ein offenes Ansprechen Druck nehmen und neue Handlungsoptionen aufzeigen kann [17, 18]. Sollten keine Todeswünsche vorhanden sein, werden durch das aktive Fragen auch keine ausgelöst [19]. Die verschiedenen Erfahrungen der Teilnehmer:innen in Bezug auf den Umgang mit Suizidwünschen betonen die Notwendigkeit einer umfassenden ethischen Reflexion und Unterstützung in diesem Bereich [3]. Ein interdisziplinärer Ansatz könnte dazu beitragen, Ärzt:innen in der Betreuung von Patient:innen mit Suizidwünschen zu unterstützen und ethische Herausforderungen zu bewältigen [20, 21]. Die zurückhaltende Einstellung gegenüber der Suizidassistenz in dieser Kohorte deutet auf eine starke Betonung von Suizidprävention und palliativer Versorgung hin. Dies entspricht den Erkenntnissen einer früheren Studie, die die Bedeutung einer umfassenden palliativen Versorgung als wichtige suizidpräventive Maßnahmen betonte [22]. Diese Ergebnisse unterstreichen die Bedeutung von Schulungs- und Weiterbildungsmaßnahmen, um Ärzt:innen bei der angemessenen Beratung und Betreuung von Patient:innen mit Suizidwünschen zu unterstützen [1, 23].

### Limitationen

Im Vergleich zu anderen Befragungen fiel die Beteiligung bei der vorliegenden deutlich geringer aus. Wir erzielten nur eine Teilnahmequote von 3 % bei insgesamt 25.573 erreichbaren Mitgliedern. Wassiliwizky et al. erreichten eine Teilnahmequote von 22 %, Schildmann et al. 20,8 %. Trotz wiederholter Dissemination der Befragung über den Newsletter der DGAI war eine größere Beteiligung nicht zu erreichen. Die erhobenen Daten können daher nur als Trend interpretierbar sein und liefern keine definitive Abbildung der Haltungen und des Wissens der Mitglieder der DGAI. Aufgrund datenschutzrechtlicher Vorgaben war es nicht möglich, eine genaue Darstellung der demografischen Daten aller Mitglieder der DGAI zur Ermittlung der Repräsentanz dieser Befragung wiederzugeben. Die angegebenen Daten wurden aus öffentlichen Mitteilungen der DGAI extrahiert und mit Quellen belegt. Aufgrund einer berufserfahreneren Kohorte sind statistische Analysen der Einflussfaktoren Alter und Berufserfahrung nur bedingt verwertbar. Weiterhin waren professionsbezogene Dependenzanalysen aufgrund eines 98%igen Anteils von Ärzt:innen nicht möglich. Aufgrund unvollständiger Datensätze konnten nicht alle abgegebenen Antworten in die statistische Berechnung aufgenommen werden. Dies kann zu einer Verzerrung des Meinungsbildes führen. Weiterhin kann eine Verzerrung vorliegen, da davon auszugehen ist, dass v. a. Mitglieder, die sich für das Thema interessierten und umfassender informiert waren, an der Befragung teilnahmen.

### Ausblick

Die Befragung zur Suizidassistenz unter Mitgliedern der DGAI unterstreicht die Komplexität dieses Themas. Zukünftige Forschung und politische Maßnahmen sollten darauf abzielen, die Bedürfnisse und Herausforderungen von Ärzt:innen und Patient:innen angemessen zu berücksichtigen und die Entwicklung klarer Richtlinien und rechtlicher Rahmenbedingungen voranzutreiben, um eine sichere und ethisch verantwortungsvolle Versorgung zu gewährleisten.

## Fazit für die Praxis

- Unter teilnehmenden Anästhesist:innen herrschen Wissenslücken im Bereich der gültigen Rechtslage zur Suizidassistenz in Deutschland.
- Die Teilnehmer:innen sprachen Patient:innen selten aktiv auf das Vorhandensein von Todeswünschen an.
- 77 % der teilnehmenden DGAI-Mitglieder wünschen sich neue gesetzliche Regelungen, um mehr Rechtssicherheit im Bereich der Suizidassistenz zu erfahren.
- 71 % der Teilnehmer:innen lehnen eine Mitwirkung beim assistierten Suizid unabhängig des Gesundheitszustands ab; 65 % können sich dies nur in palliativen Behandlungssituationen vorstellen.
- Es gibt Hinweise darauf, dass Berufserfahrung die persönliche Einschätzung und Meinung zur Suizidassistenz beeinflusst.
- Teilnehmer:innen erachten v. a. eine palliativmedizinische Versorgung als Basis suizidpräventiver Maßnahmen.

## Data Availability

Die in dieser Studie erhobenen Datensätze können auf begründete Anfrage beim Korrespondenzautor angefordert werden.
